# Inferring Past Pesticide Exposures: A Matrix of Individual Active Ingredients in Home and Garden Pesticides Used in Past Decades

**DOI:** 10.1289/ehp.9538

**Published:** 2006-11-07

**Authors:** Joanne S. Colt, Mancer J. Cyr, Shelia H. Zahm, Geoffrey S. Tobias, Patricia Hartge

**Affiliations:** 1 Division of Cancer Epidemiology and Genetics, National Cancer Institute, National Institutes of Health, Department of Health and Human Services, Bethesda, Maryland, USA; 2 Kline & Company, Inc., Little Falls, New Jersey, USA

**Keywords:** Exposure assessment, herbicides, insecticides, pesticides, residential

## Abstract

**Background:**

In retrospective studies of the health effects of home and garden pesticides, self-reported information typically forms the basis for exposure assessment. Study participants generally find it easier to remember the types of pests treated than the specific pesticides used. However, if the goal of the study is to assess disease risk from specific chemicals, the investigator must be able to link the pest type treated with specific chemicals or products.

**Objectives:**

Our goal was to develop a “pesticide–exposure matrix” that would list active ingredients on the market for treating different types of pests in past years, and provide an estimate of the probability that each active ingredient was used.

**Methods:**

We used several different methods for deriving the active ingredient lists and estimating the probabilities. These methods are described in this article, along with a sample calculation and data sources for each.

**Results:**

The pesticide–exposure matrix lists active ingredients and their probabilities of use for 96 distinct scenarios defined by year (1976, 1980, 1990, 2000), applicator type (consumer, professional), and pest type (12 categories). Calculations and data sources for all 96 scenarios are provided online.

**Conclusions:**

Although we are confident that the active ingredient lists are reasonably accurate for most scenarios, we acknowledge possible sources of error in the probability estimates. Despite these limitations, the pesticide–exposure matrix should provide valuable information to researchers interested in the chronic health effects of residential pesticide exposure.

Retrospective studies of the health effects of home and garden pesticides face challenges in exposure assessment, particularly for diseases with long latency periods where the relevant exposures may have occurred decades before diagnosis. Typically, self-reported information forms the basis for exposure assessment, sometimes supplemented with inventories of stored pesticide products or measurements of pesticide residues in environmental or biologic samples. Study participants have had difficulty recalling specific brand or chemical names of pesticides they have used (Bradman et al. 1997; Daniels et al. 2001; Pogoda and Preston-Martin 1997). Teitelbaum (2002) suggested that people can more easily remember the types of pests treated, as was observed in a study of childhood neuroblastoma (Daniels et al. 2001). If this approach is used, and if the goal is to assess disease risk from specific chemical exposures, the investigator must be able to link the type of pest treated with specific chemicals.

We have developed a “pesticide–exposure matrix” to assist in that task. The matrix is designed to be used in conjunction with self-reported information on the types of pests treated in 4 years: 1976, 1980, 1990, and 2000. For each pest–year combination, the matrix lists the active ingredients that were on the market and provides a rough estimate of the probability that a product containing each ingredient was used. For example, if a consumer treated his or her home for rodents in 1990, we estimate that there is an 87% probability that the product contained brodi-facoum, a 10% probability for warfarin, and a 3% probability for other, unspecified active ingredients. The probabilities sum to > 100% when a product contains more than one active ingredient (e.g., if only one product was available and it contained three ingredients, the probability of use would be 100% for each). The probabilities are unrelated to the concentrations of the active ingredients in the product and are therefore unrelated to the intensity of exposure.

We developed the matrix for a population-based case–control study of non-Hodgkin lymphoma (NHL) conducted between July 1988 and June 2000 (Hartge et al. 2005). Participants completed a lifetime residential history calendar and were later interviewed. Starting with the current home, interviewers asked whether pesticides were used to treat each of 12 pest types: lawn insects, lawn weeds, outdoor plant/tree insects, outdoor plant/tree weeds, outdoor plant/tree diseases, crawling insects, flying insects, termites, fleas/ticks on pets, fleas/ticks in the home, insects on indoor plants, and rodents. As the interviewer asked about each pest type, he or she displayed a card with examples of specific pests. The interviewer asked who applied the pesticide (respondent, exterminator, someone else), how frequently, and in what form (e.g., spray, powder). This was repeated for each home in which the subject lived for at least two years, going back 30 years. The questionnaire and cards can be found at http://dceg.cancer.gov/modules/PesticideHist.pdf [National Cancer Institute (NCI) 2006a].

The pesticide–exposure matrix covers each of the 12 pest types in the NHL study. Probability estimates are provided for 4 years (1976, 1980, 1990, and 2000) and two types of appliers (consumers, using pesticides purchased at supermarkets and hardware stores; and professionals such as pest control operators and lawn services). Thus, 96 “scenarios” (12 pest types × 4 years × 2 appliers) are covered. The matrix does not cover synergists [chemicals added to products to increase potency, such as N-octyl bicycloheptene dicarboximide (MGK 264) and piperonyl butoxide], repellents [e.g., DEET (diethyl-toluamide)], solvents, emulsifiers, spreaders, stickers, buffering agents, or other ingredients that are not considered active ingredients but must be listed on the label.

## Data Sources

Reports prepared by Kline & Company, Inc. (Little Falls, NJ) (Anonymous 1982, 1991; Cyr and Dansbury 2000; Fugate and Cyr 1997; Fugate and Hall 2002; Fugate et al. 2000, 2001, 2002; Garushenko et al. 1977; Goodbread et al. 1983; Hall and Dansbury 2000; Hodge and Rafter 1991, 1992a, 1992b; Ramsey and Kollonitsch 1977) were the major information source. Since 1976, Kline has conducted proprietary analyses of product sales, market share, and active ingredient sales of home and garden pesticides and fertilizers for both the consumer and professional markets. All major pesticide manufacturers in the United States are purchasers of these reports. The data are used for market planning purposes in developing business strategies focused on the consumer and professional markets. The types of information presented in the reports vary, depending on the type of market (consumer vs. professional), the pest type, and the year (data have become more detailed over time). Data may be provided on the number of acres treated nationwide with individual products, pounds of specific active ingredients used, dollar sales of products or active ingredients, prices per pound of product, dollar sales by company, and/or main products by company.

Kline derived information for the consumer markets by analyzing sales and other data obtained primarily through telephone interviews with pesticide manufacturers or formulators. Depending on the year, interviews were held with 60–75 of 85–100 manufacturers or formulators. The accuracy of the information varies with the cooperativeness of the respondents and their knowledge of the product categories, but generally increases with the size of the market. Data on market size are believed to be within 10% of the true value for product categories with sales of $500 million or more and within 25% of the true value for smaller product categories. Data for professional markets were gathered through telephone interviews with professional pesticide applicators. This market is large, highly segmented, and diffuse. Typically, 200–300 applicator companies (or branches of major chains) were interviewed in each lawn or outdoor plant/tree segment, of a universe of 15,000–18,000, and 200–800 applicator companies were interviewed in the termite, crawling insect, and flying insect segments, of a universe of about 20,000. Data accuracy varies with the cooperativeness of the respondents, their knowledge of different product categories, the number of interviews, and end user and supplier concentration in each market segment.

Two U.S. Environmental Protection Agency (EPA) databases were used. The Pesticide Product Information System (PPIS) (U.S. EPA 2003a) contains information on pesticide products that have been registered in the United States, including registrant names and addresses, ingredients, toxicity category, product names, distributor brand names, site uses, pest uses, pesticidal type, formulation code, and registration status. A related resource is U.S. EPA’s Pesticide Product Label System (PPLS) (U.S. EPA 2003b), a collection of pesticide label images. We used these databases to estimate probabilities for consumer treatment of crawling insects, flying insects, fleas/ticks on pets, and fleas/ticks in the home, and to provide information on product formulations and application rates.

Another information source, the U.S. EPA National Home and Garden Pesticide Use Survey (Whitmore et al. 1992) (the “U.S. EPA Survey”), involved home interviews with > 2,000 households in 1990. Interviewers inventoried stored pesticide products, recorded the active ingredients on the label, and asked respondents to identify the pests on which the product had been used during the preceding year. The survey covered continuous-use products (e.g., flea/tick collars, roach/ant traps) only in a general way, and did not cover professionally applied products. Because the survey accounted only for products in storage, it likely underestimated the prevalence of products that are typically discarded after a single use (e.g., foggers). We used information from this survey as input to the probability estimates for consumer treatment of crawling and flying insects.

Several other sources were used to help identify active ingredients in products and to estimate application rates: C&P Press publications (Anonymous 1994, 1995; C&P Press 2004), Meister Publishing Company manuals (Anonymous 1999a,1999b, 2000, 2003, 2005), Crop Data Management Systems, Inc. (2004), and Hagan et al. (1993).

## Methods for Estimating Probabilities and Confidence Levels

We used several different methods to estimate the probabilities. Our choice of a method for each scenario was based on the types and quality of information available. Professional judgment (M.J.C., Senior Associate, Specialty Pesticides, Kline & Company, Inc.) played a large role in many scenarios.

Wherever possible, we tied the probabilities to Kline-reported information on the number or percent of acres, nationwide, that were treated with specific products or active ingredients. That is, if Kline reported that half of all lawn acres treated for weeds by professionals was treated with active ingredient X, we assumed that if a person hired a professional to treat his or her lawn for weeds, there is a 50% chance that the applicator used a product containing X. If acreage was not provided by Kline, we attempted to derive it; otherwise, we based the probabilities on dollar sales. The probabilities were never based strictly on the pounds used, which can be a poor indicator of the probability of use; this is illustrated in Table 1, comparing two leading products used by professionals to treat for lawn insects in 2000. The pounds data erroneously suggest that Dursban (containing chlorpyrifos) was much more widely used than Talstar (containing bifenthrin), whereas the sales and acres data show that use was somewhat similar. This is because the bifenthrin molecule is more active than chlorpyrifos, and although the products sell for a similar price per acre, bifenthrin is less concentrated then chlorpyrifos in the formulated product. Overall, acreage treated probably provides the best basis for calculating probabilities because it accounts for differences in concentrations and usage rates among different classes of pesticides. We consider dollar sales to be acceptable if it is the only information available.

Our level of confidence in the probability estimates varies by scenario, depending on the method used, the extent to which judgment played a role, the quality of the data in the source materials (we occasionally judged the source data to be of poor quality and made modifications based on professional expertise), and how closely the pest type definition in the Kline reports matched that in the NHL questionnaire. Our confidence level is higher for scenarios in which one supplier or active ingredient dominated the market.

The methods used, the scenarios to which each applies, and the confidence ratings are summarized in Table 2 and discussed below. A sample calculation is provided for each (Tables 3–9). Calculations and data sources for all 96 scenarios are provided online at http://dceg.cancer.gov/pesticide (NCI 2006b).

### Method 1: number of acres treated

This method was used when the Kline reports provided the number of acres nationwide treated with specific pesticide products (scenarios 51, 52, 55, 56, 59, 60, 63, 64, and 68, all of which involve professional treatment of lawns or outdoor plants/trees). We assumed that the probability that a product (and each active ingredient in it) was used is equal to the percent of acres treated with that product.

We have a medium confidence level in the estimates for outdoor plant/tree insects (1990 (2000) and a high confidence level for the others, because the Kline data for outdoor plants do not include mature trees, which are often sprayed with insecticides by professional applicators. This use might be significant but is likely smaller than the market for insecticides applied to gardens and landscaping areas, on which the Kline estimates are based. We do not believe this to be an important limitation for outdoor plant/tree pests other than insects.

### Method 2: number of acres treated (derived from pounds of active ingredients and application rates)

This method was used for lawns and outdoor plants/trees when Kline reported the pounds of individual active ingredients sold (scenarios 2–4, 6–8, 14–16, 18–20). We divided the pounds of each active ingredient by an estimated application rate (pounds per acre) to derive the number of acres treated with each active ingredient, and then proceeded as in method 1. The application rates were taken from Meister Publishing Company manuals (Anonymous 1999a, 1999b, 2000, 2003, 2005), C&P Press publications (Anonymous 1994, 1995; C&P Press 2004), and the PPLS (U.S. EPA 2003b). If the rates were presented as a range, we chose the midpoint unless we had reason to believe otherwise.

Judgment was used for all of these scenarios, and we have a medium level of confidence in the probability estimates. For lawn weeds (1980, 1990, 2000), we modified the Kline-reported pounds of some active ingredients to reflect our judgment about actual product formulations. For lawn and outdoor plant/tree insects (1980, 1990, 2000), Kline provided the active ingredient pounds aggregated across three pest types (lawn insects, outdoor plant insects, and nonplant insects), requiring us to allocate the pounds to each individual pest type. A similar situation was encountered for outdoor plant/tree diseases (1980, 1990, 2000).

### Method 3: number of acres treated (derived from dollar sales, unit prices, and application rates)

This approach was used when Kline reported both dollar sales and unit prices (dollars per pound or gallon) for individual products (scenarios 11 and 12). We divided the dollar sales by the unit price to estimate the pounds or gallons of each product sold. We then divided the pounds or gallons sold by an estimated application rate (pounds or gallons per acre) to derive the number of outdoor plant/tree acres treated with each product, and proceeded as in method 1. We have a medium level of confidence in the estimates.

### Method 4: product sales

For scenarios 45–50, 53–54, 57–58, 61–62, 65–66, 73–76, 80, and 88–96, Kline reported dollar sales for individual products or active ingredients, but not unit prices. We assumed that the probability that a product (and each active ingredient in it) was used is equal to the product’s proportion of total dollar sales.

For 1976, Kline treated two of the NHL pest types as one (professional treatment of lawn insects and outdoor plant/tree insects were combined, as were lawn weeds and outdoor plant/tree weeds, and lawn diseases and outdoor plant/tree diseases). We allocated active ingredient sales to the individual pest types using judgment, guided by information from the Kline reports.

None of the Kline reports contained a category for professional treatment of “crawling insects” or “flying insects.” We used the following pest types reported by Kline to represent the crawling insect category: general pests in 1976 (these consist mainly of ants, roaches, and spiders), general pests and outdoor pests in 1980 (outdoor pests are mainly ants, roaches, and spiders treated outside, but not on the lawn or garden), and ants plus cockroaches in 1990 and 2000. Kline data on professional treatment of flying insects in 2000 pertained only to bees.

Because of the uncertainty associated with using product sales as the basis for probability of use, we have a medium confidence level in the probabilities for most of these scenarios. We gave high confidence ratings to the 1990 and 2000 professional termite scenarios, the former because the market was well understood by Kline and the latter because of the large sample size used by Kline. The consumer rodent market was rated high because it has been dominated by d-Con (warfarin) during the entire period of interest, and the professional segment was high because it has used a small number of active ingredients in a well-documented market. Our confidence level is low for professional treatment of outdoor plant/tree insects in 1976 and 1980 because the Kline data excluded insecticide applications to mature trees, and because the 1976 data were aggregated across more than one pest type. We have low confidence in the probabilities for flying insects in 2000 because they were based only on bees.

### Method 5: product sales (calculated from company sales)

For scenarios 1, 9, 10, 22, and 23, Kline reported dollar sales by manufacturer (but not by product or active ingredient) and identified each manufacturer’s main products and (typically) each product’s main active ingredients. Unit prices were not given. We identified the active ingredients in each product when necessary. We apportioned each manufacturer’s dollar sales to its individual products or active ingredients using judgment. We assumed that the probability that a product (and each active ingredient in it) was used is equal to the product’s percent of total dollar sales.

For indoor plants in 1990, Kline reported sales by manufacturer and we used the PPIS (U.S. EPA 2003a) to identify the active ingredients that each manufacturer might have used. Probabilities for indoor plants pertain to insects only.

Our confidence level is medium for all scenarios except lawn weeds (1976) and outdoor plant/tree weeds (1976), which are rated low because our allocation of sales to active ingredients required more judgment than the other scenarios.

### Method 6: active ingredient frequencies from PPIS

For consumer treatment of household insects (scenarios 25–40), the Kline reports were not sufficiently detailed for our purposes. We used data from the PPIS (U.S. EPA 2003a) and the U.S. EPA Survey (Whitmore et al. 1992) to estimate the active ingredient probabilities.

We first selected the PPIS “site” codes (places that the product was registered to be applied) and “pest” codes (pests that the product was registered to treat) to use for searching the database. For fleas/ticks on pets, we used site codes corresponding to pets, dogs, and cats, and pest codes for fleas, ticks, deer ticks, lonestar ticks, and brown dog ticks. For the remaining scenarios, we used the site codes listed under “household or domestic dwellings,” excluding codes that were not relevant (e.g., hotels, military barracks). For pest codes, we used cockroaches, ants, and spiders to represent crawling insects; and flies, mosquitoes, and bees to represent flying insects. We used these site and pest codes to search PPIS to identify all products that were actively registered for each pest type/year combination, excluding products that may be applied only by certified pest control operators, and the active ingredients in those products. We divided the number of products containing each active ingredient by the total number of products registered for that pest/year to derive the percent of products containing that active ingredient.

We considered setting the probability for each active ingredient equal to the percent of products in which it was contained, but this could overstate probabilities for active ingredients present in a large number of products with relatively low sales, and vice versa. We therefore used judgment to modify the probabilities for some active ingredients. We used information from Kline and the U.S. EPA Survey (Whitmore et al. 1992) as the basis for most of the modifications, and judgment for the others. Although the U.S. EPA Survey data correspond to only 1 year (1990), we assumed that the adjustments that we derived from the data would, in most instances, apply to all 4 years of interest. The U.S. EPA Survey did not provide relevant information for fleas/ticks in the home.

For fleas/ticks on pets in 2000, we also incorporated information from two Kline reports (Fugate and Cyr 1997; Fugate et al. 2001), which cover newer flea and tick products sold by veterinarians to consumers. Four veterinary products based on four active ingredients comprised 62% of this market in 2000, with products sold through retail channels comprising the remainder. We used PPIS to characterize the retail market, but used judgment based on Kline data to estimate the active ingredient probabilities for veterinary products.

The Kline reports do not cover professional treatment of fleas/ticks on pets (scenarios 81–84) because the users are typically pet grooming shops, kennels, or veterinarian offices. The products used are likely similar to those used by consumers, so we set the probabilities the same as for consumer products.

We have a medium level of confidence in the probability estimates.

### Method 7: professional judgment based on descriptive data

For scenarios 5, 13, 17, 21, and 67, probabilities were based mostly on judgment, sometimes with a small amount of quantitative and/or descriptive data from the Kline reports, the literature, and the PPLS (U.S. EPA 2003b). For indoor plants, the estimates pertain to treatment of insects only. Our confidence level is low.

### Method 8: active ingredients listed, probabilities not estimated

Kline does not maintain data on scenarios 24, 77–79, and 85–87, and there was not enough information from other sources to support probability estimates. Therefore, we developed lists of likely active ingredients but did not estimate probabilities. The lists were based on information from the Kline reports, the PPLS (U.S. EPA 2003b), the literature, and judgment.

### Method 9: no active ingredients listed or probabilities estimated

Consumer treatment of termites (scenarios 41–44) is not covered in the Kline reports. There is no evidence that a significant number of consumers purchased products to self-apply termiticides until the late 1990s, and the market remains extremely small. Indoor plants (scenarios 69–72) are rarely treated by professional applicators.

## Discussion

We describe here a pesticide–exposure matrix to assist in the assessment of exposure to individual active ingredients used in residential pesticides in the past. When used in conjunction with self-reported information on the types of pests treated in the home and garden over time, the matrix can be used to identify the active ingredients that were on the market for that pest type, and to provide a rough estimate of the probability that specific active ingredients were used.

Identifying which active ingredients a person likely used is a necessary step in exposure assessment. However, many factors that are not covered by the matrix are important determinants of a person’s level of exposure. The probabilities of use are unrelated to the concentrations of the active ingredients in the pesticide products and therefore cannot be used to infer the intensity of exposure, an important factor in assessing risk. Many other factors may influence exposure, such as the pesticide application method and location; whether the pesticide was applied by the subject or a third party; the chemical properties of the active ingredient; the presence of synergists in the product, which could affect uptake through the skin; and the use of personal protective equipment. The matrix does not address exposures from the diet, from pesticide applications at nearby homes or farms, or from community spraying programs.

Although we are confident that the active ingredient lists are reasonably accurate for most scenarios, there are many possible sources of error in the probability estimates. First, the data presented in the Kline reports were based on interviews with pesticide manufacturers and formulators or professional applicators, and the accuracy of the information depends on the number of interviews, the representativeness of the sample, the knowledge and cooperativeness of the respondents, and the complexity of the market. Second, some types of data (e.g., acres treated) are better surrogates for probability of use than others (e.g., dollar sales). Probabilities based on the percent of registered products [from the PPIS (U.S. EPA 2003a)] likely overstate probabilities for active ingredients present in a large number of products with relatively low sales, and vice versa; we attempted to correct for this, when possible, using data from the U.S. EPA Survey (Whitmore et al. 1992), but these data cover only one point in time. Third, only qualitative information was available for some scenarios. In short, a considerable amount of professional judgment was used to derive the probabilities for many scenarios. Given the many sources of uncertainty, the probabilities should be viewed as rough estimates of the relative importance of different active ingredients in each scenario.

Although we are unable to quantify the uncertainties in the probability estimates, we do provide a relative ranking of confidence levels. Of the 96 scenarios, we have a relatively high level of confidence in 17, a medium level in 54, and low confidence in 10 (mostly for 1976). For 7 scenarios, we listed the active ingredients but could not estimate probabilities. For eight scenarios we were unable to identify the active ingredients, but these scenarios are seldom encountered (homeowner treatment of termites and professional treatment of indoor plants).

Other limitations of the matrix are that it does not cover many substances present in pesticide products, such as synergists and “inert” ingredients, which may have adverse health effects. It does not incorporate information on product form because the source materials were not sufficiently detailed. Because the source data were national in scope, the matrix does not account for regional variations in pesticide use patterns. For a small number of scenarios there is a large “other” category, reflecting the level of detail in the source materials. Finally, this overall approach for assessing past pesticide use is contingent on study participants’ recall of pests treated in past homes, the accuracy of which becomes more questionable as one goes further back in time.

We know of no other source of published historical information on individual active ingredients in home and garden pesticides. Despite the noted limitations, the pesticide–exposure matrix should provide valuable information to epidemiologists and other researchers interested in the chronic health effects of residential pesticide exposure.

## Figures and Tables

**Table 1:** Comparison of two leading products used by professional applicators to treat lawns for insects in 2000.

Brand	Active ingredient	Chemical family	Sales (US$)	Acres treated	Active ingredient (lb)
Talstar	Bifenthrin	Pyrethroid	10 million	276,000	31,000
Dursban	Chlorpyrifos	Organophosphate	8 million	289,000	309,000

Data from Fugate et al. (2001).

**Table 2:** Methods used to estimate probabilities of use of specific pesticide active ingredients, and level of confidence in probability estimates.

Scenario	Pest/applier/year	Method^a^	Confidence level
1	Lawn weeds, consumer, 1976	5	Low
2	Lawn weeds, consumer, 1980	2	Medium
3	Lawn weeds, consumer, 1990	2	Medium
4	Lawn weeds, consumer, 2000	2	Medium
5	Lawn insects, consumer, 1976	7	Low
6	Lawn insects, consumer, 1980	2	Medium
7	Lawn insects, consumer, 1990	2	Medium
8	Lawn insects, consumer, 2000	2	Medium
9	Outdoor plant and tree weeds, consumer, 1976	5	Low
10	Outdoor plant and tree weeds, consumer, 1980	5	Medium
11	Outdoor plant and tree weeds, consumer, 1990	3	Medium
12	Outdoor plant and tree weeds, consumer, 2000	3	Medium
13	Outdoor plant and tree insects, consumer, 1976	7	Low
14	Outdoor plant and tree insects, consumer, 1980	2	Medium
15	Outdoor plant and tree insects, consumer, 1990	2	Medium
16	Outdoor plant and tree insects, consumer, 2000	2	Medium
17	Outdoor plant and tree diseases, consumer, 1976	7	Low
18	Outdoor plant and tree diseases, consumer, 1980	2	Medium
19	Outdoor plant and tree diseases, consumer, 1990	2	Medium
20	Outdoor plant and tree diseases, consumer, 2000	2	Medium
21	Indoor plants, consumer, 1976	7	Low
22	Indoor plants, consumer, 1980	5	Medium
23	Indoor plants, consumer, 1990	5	Medium
24	Indoor plants, consumer, 2000	8	[Table-fn tfn2-ehp0115-000248]—
25	Crawling insects, consumer, 1976	6	Medium
26	Crawling insects, consumer, 1980	6	Medium
27	Crawling insects, consumer, 1990	6	Medium
28	Crawling insects, consumer, 2000	6	Medium
29	Flying insects, consumer, 1976	6	Medium
30	Flying insects, consumer, 1980	6	Medium
31	Flying insects, consumer, 1990	6	Medium
32	Flying insects, consumer, 2000	6	Medium
33	Fleas/ticks on pets, consumer, 1976	6	Medium
34	Fleas/ticks on pets, consumer, 1980	6	Medium
35	Fleas/ticks on pets, consumer, 1990	6	Medium
36	Fleas/ticks on pets, consumer, 2000	6	Medium
37	Fleas/ticks in home, consumer, 1976	6	Medium
38	Fleas/ticks in home, consumer, 1980	6	Medium
39	Fleas/ticks in home, consumer, 1990	6	Medium
40	Fleas/ticks in home, consumer, 2000	6	Medium
41	Termites, consumer, 1976	9	[Table-fn tfn2-ehp0115-000248]—
42	Termites, consumer, 1980	9	[Table-fn tfn2-ehp0115-000248]—
43	Termites, consumer, 1990	9	[Table-fn tfn2-ehp0115-000248]—
44	Termites, consumer, 2000	9	[Table-fn tfn2-ehp0115-000248]—
45	Rodents, consumer, 1976	4	High
46	Rodents, consumer, 1980	4	High
47	Rodents, consumer, 1990	4	High
48	Rodents, consumer, 2000	4	High
49	Lawn weeds, professional, 1976	4	Medium
50	Lawn weeds, professional, 1980	4	Medium
51	Lawn weeds, professional, 1990	1	High
52	Lawn weeds, professional, 2000	1	High
53	Lawn insects, professional, 1976	4	Medium
54	Lawn insects, professional, 1980	4	Medium
55	Lawn insects, professional, 1990	1	High
56	Lawn insects, professional, 2000	1	High
57	Outdoor plant and tree weeds, professional, 1976	4	Medium
58	Outdoor plant and tree weeds, professional, 1980	4	Medium
59	Outdoor plant and tree weeds, professional, 1990	1	High
60	Outdoor plant and tree weeds, professional, 2000	1	High
61	Outdoor plant and tree insects, professional, 1976	4	Low
62	Outdoor plant and tree insects, professional, 1980	4	Low
63	Outdoor plant and tree insects, professional, 1990	1	Medium
64	Outdoor plant and tree insects, professional, 2000	1	Medium
65	Outdoor plant and tree diseases, professional, 1976	4	Medium
66	Outdoor plant and tree diseases, professional, 1980	4	Medium
67	Outdoor plant and tree diseases, professional, 1990	7	Low
68	Outdoor plant and tree diseases, professional, 2000	1	High
69	Indoor plants, professional, 1976	9	[Table-fn tfn2-ehp0115-000248]—
70	Indoor plants, professional, 1980	9	[Table-fn tfn2-ehp0115-000248]—
71	Indoor plants, professional, 1990	9	[Table-fn tfn2-ehp0115-000248]—
72	Indoor plants, professional, 2000	9	[Table-fn tfn2-ehp0115-000248]—
73	Crawling insects, professional, 1976	4	Medium
74	Crawling insects, professional, 1980	4	Medium
75	Crawling insects, professional, 1990	4	Medium
76	Crawling insects, professional, 2000	4	Medium
77	Flying insects, professional, 1976	8	[Table-fn tfn2-ehp0115-000248]—
78	Flying insects, professional, 1980	8	[Table-fn tfn2-ehp0115-000248]—
79	Flying insects, professional, 1990	8	[Table-fn tfn2-ehp0115-000248]—
80	Flying insects, professional, 2000	4	Low
81	Fleas/ticks on pets, professional, 1976	6	Medium
82	Fleas/ticks on pets, professional, 1980	6	Medium
83	Fleas/ticks on pets, professional, 1990	6	Medium
84	Fleas/ticks on pets, professional, 2000	6	Medium
85	Fleas/ticks in home, professional, 1976	8	[Table-fn tfn2-ehp0115-000248]—
86	Fleas/ticks in home, professional, 1980	8	[Table-fn tfn2-ehp0115-000248]—
87	Fleas/ticks in home, professional, 1990	8	[Table-fn tfn2-ehp0115-000248]—
88	Fleas/ticks in home, professional, 2000	4	Medium
89	Termites, professional, 1976	4	Medium
90	Termites, professional, 1980	4	Medium
91	Termites, professional, 1990	4	High
92	Termites, professional, 2000	4	High
93	Rodents, professional, 1976	4	High
94	Rodents, professional, 1980	4	High
95	Rodents, professional, 1990	4	High
96	Rodents, professional, 2000	4	High

— Probabilities were not estimated for these scenarios.

a 1 = number of acres treated; 2 = number of acres treated, derived from pounds of active ingredients and application rates; 3 = number of acres treated, derived from dollar sales, unit prices, and application rates; 4 = product sales; 5 = product sales, calculated from company sales; 6 = active ingredient frequencies from PPIS (U.S. EPA 2003a); 7 = professional judgment based on descriptive data; 8 = active ingredients listed, probabilities not estimated; 9 = no active ingredients listed or probabilities estimated.

**Table 3:** Example of method 1: professional treatment of outdoor plant/tree insects, 1990 (scenario 63).

Product^a^	Active ingredient	Acres treated^a^	Probability of use [% (calculated)]^b^
Malathion	Malathion	90,000	31
Dursban	Chlorpyrifos	47,000	16
Diazinon	Diazinon	32,000	11
Sevin	Carbaryl	11,000	4
Orthene	Acephate	10,000	3
Oftenol	Isofenphos	9,000	3
Other	Other	96,000	33
Total		295,000	

a Anonymous (1991).

b Acres treated with each active ingredient divided by total acres treated.

**Table 4:** Example of method 2: consumer treatment of lawn insects, 1980 (scenario 6).

	Pounds applied to lawns, outdoor plants, and nonplants^a^		Pounds applied to lawns, outdoor plants, and nonplants^b^					
Active ingredient^a^	Fertilizer/insecticide combination products^a^	Insecticide-only products^a^	Lawn	Outdoor plants	Nonplants	Application rate for lawns (lbs/acre)^c^	Lawn acres treated (calculated)^d^	Probability of use for lawns [% (calculated)]^e^
Diazinon	1,200,000	800,000	1,400,000	480,000	120	3	467,000	63
Chlorpyrifos	140,000	110,000	190,000	44,000	17	2	95,000	13
Carbaryl	0,000	650,000	33,000	520,000	98	3	11,000	1
Malathion	0,000	400,000	20,000	320,000	60	2	10,000	1
Other	230,000	590,000	319,000	413,000	89	2	159,000	21
Total	1,570,000	2,550,000	1,961,000	1,777,000	383		742,000	

a Anonymous (1982).

b Allocation of active ingredient pounds separately to lawns, outdoor plants, and nonplants was done as follows: fertilizer/insecticide combination products: 100% is applied to lawns (judgment). Insecticide-only products: 15% of the total is applied to lawns, 70% to outdoor plants, and 15% to nonplants (Anonymous 1982). The split of each active ingredient to lawns vs. outdoor plants vs. nonplants is based on judgment, using the following assumptions: diazinon: 25%–60%–15%, carbaryl and malathion: 5%–80%–15%, chlorpyrifos: 45%–40%–15%, other: 15%–70%–15%.

c Meister Publishing Company manuals (Anonymous 1999a, 1999b, 2003).

d Pounds applied to lawns divided by application rate for lawns.

e Lawn acres treated with each active ingredient divided by total lawn acres treated.

**Table 5:** Example of method 3: consumer treatment of outdoor plant/tree weeds, 1990 (scenario 11).

Manufacturer/ product^a^	Sales (US$ million)^a^	Unit price ($/gal)^a^	Gal used [million (calculated)]^b^	Application rate (gal/acre)^c^	Acres treated [million (calculated)]^d^	Active ingredient	Acres treated [million (calculated)]^e^	Active ingredient^f^	Acres treated [million (calculated)]^g^	Probability of use [%(calculated)]^h^
Monsanto	90.0	48	1.9	0.5	3.8	Glyphosate	3.8	Glyphosate	4.0	42
Chevron Ortho								2,4-D	2.2	23
Kleenup	7.0	60	0.1	0.5	0.2	Glyphosate	0.2	MCPP	2.2	23
Weed-b-Gone	20.0	32	0.6	0.4	1.6	2,4-D MCPP	1.6 1.6	Diquat Dacthal	1.0 1.0	11 11
Triox	4.3	24	0.2	0.5	0.4	Prometon	0.4	Trifluralin	1.0	10
Lebanon	3.5	18	0.2	0.2	1.0	Trifluralin	1.0	Prometon	0.4	4
VPG Fertilome	2.1	24	0.1	0.4	0.2	2,4-D MCPP	0.2 0.2			
Kmart	2.0	24	0.1	0.4	0.2	2,4-D MCPP	0.2 0.2			
Spectracide	2.0	24	0.1	0.4	0.2	2,4-D MCPP	0.2 0.2			
Other products	16.5	20	0.8	0.4	2.1	Dacthal Diquat	1.0 1.0			
Total					9.6					

Abbreviations: 2,4-D, 2,4-dichlorophenoxyacetic acid; MCPP, mecoprop.

a Hodge and Rafter (1992a).

b Sales divided by unit price.

c Meister Publishing Company manuals (Anonymous 1999a, 1999b, 2003), C&P Press publications (Anonymous 1994, 1995; C&P Press 2004).

d Gallons used divided by application rate in gallons per acre.

e Assigning each product’s acres treated to all of the active ingredients it contains.

f Eliminating duplicates.

g Combining active ingredient acres treated across all products in which it appears.

h Dividing each active ingredient’s acres treated by the total number of acres treated.

**Table 6:** Example of method 4: professional treatment of fleas/ticks in the home, 2000 (scenario 88).

Product^a^	Active ingredient	Sales (US$)^a^	Active ingredient^b^	Sales (US$)^c^	Probability of use [% (calculated)]^d^
Archer IGR	Pyridine	355,000	Methoprene	3,371,000	26
Catalyst	Propetamphos	2,528,000	Propetamphos	2,528,000	19
Demand CS	Lambda-cyhalothrin	393,000	Permethrin	1,296,000	10
Demon	Cypermethrin	163,000	Chlorpyrifos	1,083,000	8
Diazinon	Diazinon	166,000	Deltamethrin	794,000	6
Dragnet SFR	Permethrin	475,000	Pyriproxifen	550,000	4
Dursban 50W	Chlorpyrifos	425,000	Bendiocarb	486,000	4
Dursban Pro	Chlorpyrifos	658,000	Lambda-Cyhalothrin	393,000	3
Ficam W	Bendiocarb	486,000	Diazinon	371,000	3
Flee	Permethrin	660,000	Pyridine	355,000	3
Lindane	Lindane	118,000	Tralomethrin	244,000	2
Nylar IGR	Pyriproxifen	134,000	Cypermethrin	163,000	1
Nylar	Linalool	118,000	Cyfluthrin	122,000	1
Precor 2000	Methoprene	817,000	Lindane	118,000	1
Precor IGR	Methoprene	2,189,000	Linalool	118,000	1
Precor IGR	Methoprene	365,000	Other	1,094,000	8
Prelude	Permethrin	161,000			
Saga	Tralomethrin	244,000			
Suspend	Deltamethrin	794,000			
Tempo	Cyfluthrin	122,000			
Ultracide aerosol	Pyriproxifen	416,000			
Diazinon 4E	Diazinon	205,000			
Other	Other	1,094,000			
Total		13,086,000			

a Fugate et al. (2000).

b Eliminating duplicates.

c Combining active ingredient sales across all products in which it appears.

d Sales for each active ingredient divided by total sales.

**Table 7:** Example of method 5: consumer treatment of indoor plants (insects only), 1990 (scenario 23).

Manufacturer^a^	Sales (US$)^a^	Active ingredient^b^	Sales (US$)^c^	Active ingredient^d^	Sales (US$)^e^	Probability [%(calculated)]^f^
Safer	1,344,000	Fatty acids	1,344,000	Pyrethrins	2,306,000	32
Ortho (Chevron)	1,000,000	Acephate	500,000	Resmethrin	2,050,000	28
		Resmethrin	500,000	Fatty acids	1,706,000	24
Hyponex (Scotts)	800,000	Pyrethrins	800,000	Phenothrin	1,164,000	16
		Resmethrin	800,000	Allethrin	1,144,000	16
Dexol	641,000	Dysiston	641,000	Dysiston	786,000	11
SC Johnson RAID	1,500,000	Pyrethrins	750,000	Tetramethrin	769,000	11
		Allethrin	750,000	Acephate	645,000	9
		Tetramethrin	375,000	Permethrin	145,000	2
		Resmethrin	750,000	Other	290,000	4
		Phenothrin	375,000			
United	1,183,000	Pyrethrins	394,000			
		Allethrin	394,000			
		Tetramethrin	394,000			
		Phenothrin	789,000			
Other	746,000	Fatty acids	362,000			
		Pyrethrins	362,000			
		Acephate	145,000			
		Dysiston	145,000			
		Permethrin	145,000			
		Other	290,000			
Total	7,214,000					

a Hodge and Rafter (1992a).

b PPLS (U.S. EPA 2003b).

c Assignment of dollar sales to individual active ingredients was based on the PPLS (U.S. EPA 2003b) and judgment.

d Eliminating duplicates.

e Combining active ingredient sales across all manufacturers that produce it.

f Sales of each active ingredient divided by total sales.

**Table 8:** Example of method 6: consumer treatment of crawling insects, 2000 (scenario 28).

Active ingredient^a^	No. of products^a^	Probability [%(calculated)]^b^	Probability [%(adjusted)]
Permethrin	436	17.1	17
Pyrethrins	746	29.3	15^c^
Chlorpyrifos	321	12.6	13
Allethrin	250	9.8	10
Propoxur	80	3.1	9^c^
Diazinon	213	8.4	8
Tetramethrin	190	7.5	7
Hydramethylnon	15	0.6	8^d^
Fipronil	12	0.5	8^d^
Dichlorvos	52	2.0	6^c^
Sulfluramid	8	0.3	6^e^
Phenothrin	136	5.3	5
Resmethrin	213	8.4	4^c^
Boric acid	89	3.5	3
Carbaryl	80	3.1	3
Pyriproxifen	70	2.7	3
Esfenvalerate	68	2.7	3
Cyfluthrin	56	2.2	2
Deltamethrin	41	1.6	2
Fenvalerate	40	1.6	2
Malathion	39	1.5	2
Methoprene	9	0.4	2^e^
Hydroprene	8	0.3	2^c^
Prallethrin	29	1.1	1
Cypermethrin	28	1.1	1
Eugenol	10	0.4	1^e^
Other	196	7.7	8
Total	2,546		

a From analysis of PPIS (U.S. EPA 2003a) data. The numbers do not sum to the total number of products because many products contain more than one active ingredient.

b Number of products containing each active ingredient divided by the total number of products.

c We modified the probability based on information on treatment of cockroaches, ants, and spiders from the U.S. EPA Survey (Whitmore et al. 1992).

d Based on information from Kline (Hall and Dansbury 2000; Fugate and Hall 2002) and judgment.

e We modified the probability based on judgment.

**Table 9:** Example of method 7: consumer treatment of outdoor plant/tree diseases, 1976 (scenario 17).

Active ingredient	Probability (%)^a^
Captan	20
Folpet	20
Sulfur	20
Chlorothalonil	15
Maneb	10
Zineb	5
Thiram	5
Ferbam	5

a According to Kline (Ramsey and Kollonitsch 1977), Ortho was the largest manufacturer in this segment, with a 33% market share. Ortho’s main active ingredients were captan, folpet, and sulfur, which were used by other manufacturers as well. Other active ingredients listed by Ramsey and Kollonitsch (1977), and most likely used by both Ortho and other manufacturers, were chlorothalonil, maneb, zineb, thiram, and ferbam. Based on this information, we used judgment to derive the probabilities.
